# The impacts of recent smoking control policies on individual smoking choice: the case of Japan

**DOI:** 10.1186/2191-1991-3-4

**Published:** 2013-03-08

**Authors:** Michio Yuda

**Affiliations:** 1 School of Economics, Chukyo University, 101-2 Yagoto-honmachi, Showa-ku, Nagoya, 4668666, Japan

**Keywords:** Smoking, Cigarette tax/price increase, The health promotion law, Instrumental variable probit model, Japan, C25, C26, I18

## Abstract

**Abstract:**

This article comprehensively examines the impact of recent smoking control policies in Japan, increases in cigarette taxes and the enforcement of the Health Promotion Law, on individual smoking choice by using multi-year and nationwide individual survey data to overcome the analytical problems of previous Japanese studies. In the econometric analyses, I specify a simple binary choice model based on a random utility model to examine the effects of smoking control policies on individual smoking choice by employing the instrumental variable probit model to control for the endogeneity of cigarette prices. The empirical results show that an increase in cigarette prices statistically significantly reduces the smoking probability of males by 1.0 percent and that of females by 1.4 to 2.0 percent. The enforcement of the Health Promotion Law has a statistically significant effect on reducing the smoking probability of males by 15.2 percent and of females by 11.9 percent. Furthermore, an increase in cigarette prices has a statistically significant negative effect on the smoking probability of office workers, non-workers, male manual workers, and female unemployed people, and the enforcement of the Health Promotion Law has a statistically significant effect on decreasing the smoking probabilities of office workers, female manual workers, and male non-workers.

**JEL classification:**

C25, C26, I18

## Background

It is known that smoking causes serious health problems, not only for smokers but also for non-smokers through second-hand smoke (for example, The International Bank for Reconstruction and Development/ The World Bank [1]). To reduce health damage from smoking, the World Health Organization (WHO) ratified *The Framework Convention on Tobacco Control* in 2003, and many developed countries have implemented a variety of smoking control policies. In fact, various smoking restrictions are enforced in many European countries^a^, and individual states in the U.S. have imposed smoking restrictions.

**Figure 1:**
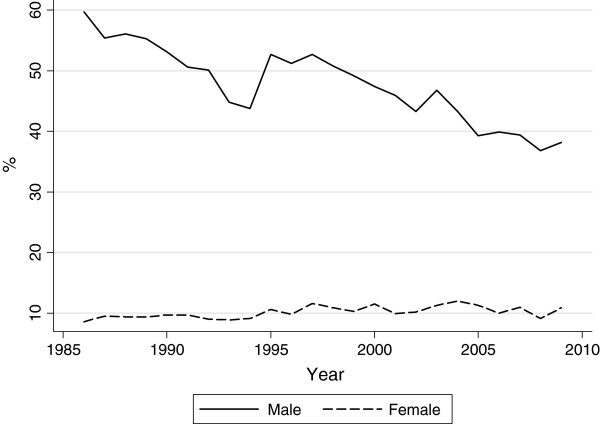
**Smoking rates in Japan. Source:** The National Nutrition Survey (1986–2002) and The National Nutrition and Health Survey (2003–2009), the Ministry of Health, Labour and Welfare. **Note:** The definitions of a current smoker differ by survey years. **(i) 1986–1989:** a current smoker is defined as a respondent who chooses “Yes” in response to the question “Do you smoke?”. **(ii) 1990–2002:** a respondent who chooses “Yes, I am a current smoker.” in response to the same question. **(iii) 2003–2009:** a respondent who smoked every day or most days of the week during the month just before the survey and has smoked more than 100 cigarettes or for more than six months.

In contrast, the Japanese government has only recently begun to take measures to decrease the smoking rate to that of other developed countries and to reduce medical expenditures for smoking-related diseases. Specifically, the Japanese government formulated *The National Health Promotion in the 21st Century Initiative (Health Japan 21)* in March 2000, ratified the WHO’s tobacco control convention in June 2006, and enforced several smoking control policies in recent years. As shown in Figure 1, the smoking participation rate of females has remained steady at approximately 10 percent, whereas that of males has decreased by approximately 20 percent over the last twenty-five years. As can be inferred from *The National Nutrition Survey* and *The National Nutrition and Health Survey* of the Ministry of Labour, Health, Welfare, the trend of females is thought to be due to both an increase in the smoking rates of the young and a decrease in those of the aged, and that of males is due to a decrease in the smoking rates of each generation, especially among young people. In particular, the smoking rates of men dropped suddenly in the early years of this century. This tendency may reflect the effects of recent efforts to control smoking.

The establishment of policy objectives for smoking rates and the design of future smoking control policies require the assessment of current systems. In fact, numerous studies have examined the effects of an increase in cigarette taxes as well as the effects of smoking control policies on smoking behavior. Chaloupka and Warner [2] and Cawley and Ruhm [3] comprehensively summarize these studies and show that increases in cigarette taxes and the implementation of smoking bans contribute to reducing smoking rates in many countries. In Japan, many researchers have empirically examined the effect of smoking control policies on smoking behavior and the demand for cigarettes (Sato and Ohkusa [4]; Kadota et al. [5]; Ogura et al. [6]; Kotani, et al. [7,8]; Ishii and Kawai [9]; Morozumi and Ii [10]; Kamimura and Noda [11]; and Ueda et al. [12])^b^. Some studies have found that both the demand for cigarettes and the probability of smoking are statistically significantly reduced by an increase in cigarette prices (Kadota et al. [5] and Kamimura and Noda [11]) and by implementing smoking regulations at home, in the office, and in public spaces (Ogura et al. [6] and Morozumi and Ii [10]). However, these Japanese studies have at least two serious econometric problems that lead to incorrect estimations of the effects of smoking control policies on cigarette smoking. The first problem is that most of these studies have used a single-year cross-sectional dataset. Because Japanese smoking control policies are uniformly enforced nationwide, it is difficult to distinguish between the effects of smoking control policies and yearly effects (time trends) when using a single-year cross-sectional dataset. The second problem is that these studies have not sufficiently discussed the endogeneity of the smoking bans. Evans, Farrelly, and Montgomery [13] notes that smoking bans and restrictions in public places can generate the potential for self-selection bias. Therefore, it is necessary to thoroughly consider their endogeneity to estimate the true effect of smoking bans.

The advantages of this study, which uses multi-year and nationwide individual survey data, are that it overcomes the above analytical problems of previous Japanese studies and comprehensively examines the impacts of recently implemented smoking control policies in Japan on individual smoking choice. With regard to the former, I can partially distinguish between the effects of the implementation of smoking control policies and other yearly effects by using a multiple-year dataset. In addition, the use of a multiple-year dataset also enables us to estimate the long-term effects of those policies on smoking behaviour, which have not been examined by previous Japanese studies. With regard to the latter, this is the first Japanese study that comprehensively investigates the impacts of recently implemented smoking control policies. In particular, this study focuses on the effects of two main smoking control policies in Japan, increases in cigarette taxes and the enforcement of the Health Promotion Law (HPL). Tobacco taxes per cigarette have increased three times in this century: they were increased by 1 yen in July 2003 and July 2006 and by 3.5 yen in October 2010. The HPL, established in May 2003, aims to improve the nutritional status and health of the Japanese people. In particular, article 25 of the HPL is the first provision in Japan with stipulations for preventing second-hand smoke inhalation in public spaces, such as schools, gymnasiums, restaurants, hospitals, theaters, assembly halls, exhibition halls, department stores, business offices, and government and other public offices^c^.

The empirical results of this study show that an increase in cigarette prices has a statistically significant effect on the reduction of smoking probability of males by 1.0 percent and of females by 1.4 to 2.0 percent. Furthermore, the enforcement of the HPL has a statistically significant effect on the reduction of the smoking probability of males by 15.2 percent and of females by 11.9 percent. Moreover, increases in cigarette prices have a statistically significant negative effect on smoking probability for office workers and non-workers, male manual workers, and female unemployed people. The introduction of the HPL has a statistically significant effect on the decrease of smoking probabilities of office workers, female manual workers, and male non-workers.

The remainder of this paper is organized as follows. Section 2 describes the dataset. Section 3 presents the econometric models and empirical strategies. Section 4 presents the empirical results, and Section 5 concludes the paper.

## Methods

### Data^d^

The data used in this study are from *the Japanese General Social Surveys* (JGSS) for 2000, 2001, 2002, 2003, 2005, and 2006. The JGSS are nationally representative surveys designed and conducted by the JGSS Research Center at the Osaka University of Commerce (Joint Usage / Research Center for Japanese General Social Surveys accredited by the Minister of Education, Culture, Sports, Science and Technology) in collaboration with the Institute of Social Science at the University of Tokyo^e^.

A question about respondents’ habitual smoking in the JGSS is “Do you smoke?” The answers differ by year. In the surveys from 2000 and 2001, respondents chose “Yes” or “No.” After the 2002 survey, respondents chose one of the following: “I am a smoker”, “I used to smoke, but I have stopped smoking”, or “I have scarcely/ never smoked.” In this study, a smoker is defined as a current smoker who chooses “Yes” or “I am a smoker.^f^” Table 1 shows the smoking rates from all of the JGSS. Compared to Figure 1, the smoking rates from the JGSS are slightly higher than those from *The National Nutrition Survey* and *The National Nutrition and Health Survey* for both genders. These gaps may be due to differences in the question formats and examination methods (Akiyama et al. [14]^g^). However, the recent downward trend for males is also found in the JGSS sample.

**Table 1:** About the Japanese general social surveys

**Year**	**Month**	**Form**	**Number of respondents**		**Number of valid responses**	**Response rate**^**3)**^	**Smoking rates**^**4)**^		
			**Total**^**1)**^	**Regular respondents**^**2)**^			**Total**	**Male**	**Female**
1999	March	(Pilot survey, Tokyo)	380	380	159	43.8%	37.0%^5)^		
		(Pilot survey, Osaka)	374	374	151	43.3%			
1999	October -November	(Pilot survey)	1,277	1,200	790	65.0%	34.0%	56.6%	14.6%
2000	October -November		4,719	4,498	2,893	64.9%	31.5%	50.0%	15.9%
2001	October -November		4,822	4,498	2,790	63.1%	29.5%	47.3%	14.0%
2002	October -November		5,354	5,000	2,953	62.3%	28.7%	47.3%	12.8%
2003	October -November	A	4,039	3,578	1,957	55.0%	25.8%	42.9%	12.1%
	October -November	B	4,044	3,622	1,706	48.0%			
2005	August -November		4,500	4,500	2,023	50.5%	26.4%	41.6%	13.8%
2006	October -December	A	4,002	4,002	2,124	59.8%	25.1%	39.0%	12.2%
	October -December	B	3,998	3,998	2,130	59.8%			
2008	October -December	A	3,997	3,997	2,060	58.2%	24.5%	39.3%	11.0%
	October -December	B	4,003	4,003	2,160	60.6%			
2009	January -March	(Special survey)	6,000	6,000	2,727	51.1%	*Unpublished*		
2010	February -April	A	4,500	4,500	2,507	62.2%	*Unpublished*		
	February -April	B	4,500	4,500	2,496	62.1%	*Unpublished*		

Source: http://jgss.daishodai.ac.jp/english/index.html.

Note: 1) Including supplements.

2) Supplemental surveys were not used after the 2005 survey.

3) Calculated only by original sample.

4) Unpublished indicates that respondents’ habitual smoking has been surveyed but is not yet released.

5) Information on respondents’ gender and residence is not available in the first pilot survey.

### Econometric model

Based on a random utility model, I specify a simple binary choice model to examine the effects of smoking control policies on individual smoking choice:

(1) $$\begin{array}{c} \mathrm{Smokin}g_{\mathrm{it}}^{*}=\alpha_{0}+\alpha_{1}\cdot \mathrm{CigTa}x_{t}+\alpha_{2}\cdot \mathrm{HP}L_{t}\\ \;+\sum_{j=1}^{J}\beta_{j}\cdot x_{j,\mathrm{it}}+u_{\mathrm{it}} \end{array}$$

(none1) $$\mathrm{Smokin}g_{\mathrm{it}}=\{\begin{array}{l} 1\;if\;Smoking_{\mathrm{it}}^{*}\&0\\ \;0\;otherwise \end{array}$$

*Smoking* is an indicator that equals one if individual *i* is a current smoker. *CigTax* is the amount of the cigarette tax per package in year *t* adjusted to 2005 prices. *HPL* is a proxy variable that captures the enforcement of the HPL. Generally, a dummy variable that equals one if observations are after 2003 and zero otherwise is used as a proxy for the effect of the introduction of the HPL. However, because the HPL was uniformly enforced nationwide to a certain point (May 2003), this dummy variable can be expressed in a linear combination of survey year dummy variables. Thus, the use of a dummy variable for the HPL may allow us to avoid discriminating between these policy effects and each unobserved yearly effect. To cope with this problem, I also use the elapsed years of the implementation of the HPL. In other words, the HPL dummy variable captures the effect of the enforcement of the HPL (the basic model), and the variable of the elapsed years of the HPL captures its diffusion effect (the dynamic model). By using these variables, I distinguish between policy effects and unobserved year effects and consider the effects of people’s gradual cognizance of the HPL over time. If these policies have negative impacts on smoking probability, *α*_*1*_ and *α*_*2*_ are expected to be statistically significant and negative.^h^

The variable *x*_*j*_ includes individual attributes, such as the respondent’s age and its square, years of education, employment formats (including four types^i^), marital status, the number of housemates (over age 20 and under age 20), income (including eight categories^j^), size of city of residence (the 13 largest cities/ other cities), local effects (prefectural dummy variables), and yearly business cycle effects and time trends (real GDP (gross domestic product) and unemployment rate in year *t*)^k^. In addition, as mentioned in footnote C, some municipalities introduced smoking bans in the street. Because information on the respondent’s residence at the municipality level is not available from the JGSS datasets, however, I add the interaction terms between prefectural dummy variables and urban scale dummy variables on explanatory variables to control for local smoking bans at the municipality level^l^.

### Endogeneity of policy variables

It should be noted that the two policy variables (*CigTax* and *HPL*) may be endogenous in the sense of econometric theory. If I directly estimate equation (1), the parameters are biased.

Because cigarette taxes can only be changed by Japan’s central government, they have been used as an exogenous variable in previous studies. However, Japan Tobacco, Inc. (JT) additionally increased the cigarette price by 0.5 yen per cigarette in July 2006 to cover the cost of introducing new cigarette vending machines (JT [15]) and by 1.5 yen to compensate for anticipated lower revenues due to a substantial price increase in October 2010 (JT [16]). Thus, because cigarette companies engage in increasing prices, using the cigarette price as one of the independent variables generate simultaneous bias. To control for this simultaneous bias, I specify the following equations and estimate them by the instrumental variable (IV) estimation.

(2) $$\begin{array}{c} \mathrm{Smokin}g_{\mathrm{it}}^{*}=\alpha_{0}+\alpha_{1}\cdot \mathrm{CigPric}e_{t}+\alpha_{2}\cdot \mathrm{HP}L_{t}\\ \;+\sum_{j=1}^{J}\beta_{j}\cdot x_{j,\mathrm{it}}+u_{\mathrm{it}} \end{array}$$

(3) $$\begin{array}{c} \mathrm{CigPrice}=\gamma_{0}+\gamma_{H}\cdot \mathrm{HP}L_{t}\\ \;+\sum_{j=1}^{J}\gamma_{j}\cdot x_{j,\mathrm{it}}+\delta \cdot \mathrm{CigTa}x_{t}+v_{\mathrm{it}} \end{array}$$

*CigPrice* is the retail cigarette price per package in year *t* adjusted to 2005 prices and *CigTax* is an instrument of *CigPrice* in this system. Because cigarette taxes can only be changed by Japan’s central government, *CigTax* is considered exogenous and has sufficient explanatory power for *CigPrice* (for example, Keeler et al. [17]).

Smoking restrictions in public places, such as the HPL, may also be endogenous because they may generate the potential for self-selection bias. For example, Evans, Farrelly, and Montgomery [13] notes that firms and areas with many non-smokers tend to implement smoking bans, that non-smokers may be attracted to firms with workplace smoking bans and that firms with the highest level of environmental tobacco smoke are more likely to ban workplace smoking. The HPL does not have penal regulations, and individuals’ preferences regarding smoking in an area may reflect the strictness of the smoking control policies in the area. If these effects are not considered, *HPL* and the error terms may be correlated, which biases the estimators. In practice, prefectural dummy variables capture prefectural unobserved heterogeneity to consistently estimate the parameters.

To estimate equations (2) and (3) with the instrumental variable probit (IV-Probit) model, the error terms *u* and *v* are assumed (*u*_*it*_, *v*_*it*_) ∼ *N*(0, *Σ*), where var(*u*_*it*_) is one to identify the model.

### Further analysis

I additionally examine the effects of smoking control policies by employment format. Because the HPL stipulates that smoking is regulated only in public spaces, smoking behavior by employment format may differ after the enforcement of the HPL. Specifically, the smoking rates of individuals who work in places where the HPL prohibits smoking may have decreased after 2003, whereas people who work in other places and those who do not work may not have changed their behavior. In other words, I can examine the true effects of the enforcement of the HPL because this situation is deemed a natural experiment. To examine the effects of smoking control policies on each smoking behavior, I divide individuals into four employment formats^m^ and estimate the following equations with interaction terms between smoking control policies and employment format dummy variables by the two-step IV-Probit estimation^n^.

(4) $$\begin{array}{c} \mathrm{Smokin}g_{\mathrm{it}}^{*}=\alpha_{0}+\sum_{k=1}^{4}\alpha_{1k}\cdot \mathrm{CigPric}e_{t}\cdot \mathrm{Employmen}t_{k,\mathrm{it}}\\ \;+\sum_{k=1}^{4}\alpha_{2k}\cdot \mathrm{HP}L_{t}\cdot \mathrm{Employmen}t_{k,\mathrm{it}}+\sum_{j=1}^{J}\beta_{j}\cdot x_{j,\mathrm{it}}+u_{\mathrm{it}} \end{array}$$

(5) $$\begin{array}{c} \mathrm{CigPric}e_{t}\cdot \mathrm{Employmen}t_{k,\mathrm{it}}\\ \;=\gamma_{0}+\sum_{k=1}^{4}\gamma_{\mathrm{Hk}}\cdot \mathrm{Employmen}t_{k,\mathrm{it}}\cdot \mathrm{HP}L_{t}\\ \;+\sum_{j=1}^{J}\gamma_{j}\cdot x_{j,\mathrm{it}}+\sum_{k=1}^{4}\delta_{k}\cdot \mathrm{Employmen}t_{k,\mathrm{it}}\cdot \mathrm{CigTax}\\ \;+v_{it}\left(\mathrm{for}\;k=1,2,3,\mathrm{and}4\right) \end{array}$$

Four independent variables for *Employment*_*k*_ indicate the individual’s employment format: *office workers* (*k* = 1), *manual workers* (*k* =2), *the unemployed* (*k* = 3), and *non-workers* (*k* = 4).

### Estimation strategies

I estimate separate equations for gender because actual smoking rates and smoking behavior among males and females are quite different, as noted by recent studies, such as Bauer et al. [18], Stehr [19], and Lundborg and Andersson [20]. In addition, because error terms are serially correlated when multi-year repeated cross-sectional datasets are used, standard errors could be underestimated (Bertrand et al. [21]). Therefore, I estimate clustering-robust standard errors at the prefectural level, which allows correlations of disturbance among individuals who live in the same prefecture (Anglist and Pischke [22]).

## Results and discussion

### Descriptive statistics

Table 2 shows the descriptive statistics for the major variables by gender. When I drop observations that have missing values for one or more of the variables in the models, the sample size is 4367 for males and 2970 for females. Of this sample, 53.1 percent of males and 23.8 percent of females are habitual smokers. The smoking rates of both genders are slightly higher than those of the macro level, as shown in Figure 1, which should be noted in the interpretation of the following empirical results.

**Table 2:** Summary statistics of the main variables

**Gender**	**Male**				**Female**			
**Variabels**	**Mean**	**SD**	**Min**	**Max**	**Mean**	**SD**	**Min**	**Max**
Smoking (=1 if current smoker)	0.531	0.499	0.000	1.000	0.238	0.426	0.000	1.000
Cigarette price per pack ^1), 2)^	246.033	11.412	238.876	272.318	241.901	8.297	238.876	272.318
Cigarette tax per pack ^1), 3) , 4)^	146.107	13.261	135.146	174.321	140.234	9.969	135.146	174.321
Implementation of the Health Promotion Law (HPL)	0.347	0.476	0.000	1.000	0.142	0.349	0.000	1.000
Elapsed years of implementation of the HPL	0.919	1.462	0.000	4.000	0.391	1.066	0.000	4.000
Age	53.457	16.023	20.000	89.000	51.771	16.871	20.000	89.000
Years of education	12.268	2.913	6.000	18.000	11.686	2.542	6.000	18.000
Employment status								
Office worker	0.506	0.500	0.000	1.000	0.433	0.496	0.000	1.000
Manual worker	0.204	0.403	0.000	1.000	0.069	0.254	0.000	1.000
Unemployment	0.029	0.167	0.000	1.000	0.012	0.108	0.000	1.000
Non-worker (Reference group)	0.261	0.439	0.000	1.000	0.486	0.500	0.000	1.000
Income class								
0- 1 million yen (Reference group)	0.311	0.463	0.000	1.000	0.458	0.498	0.000	1.000
1- 2.5 million yen	0.084	0.277	0.000	1.000	0.150	0.357	0.000	1.000
2.5- 3.5 million yen	0.104	0.305	0.000	1.000	0.088	0.283	0.000	1.000
3.5- 4.5 million yen	0.118	0.323	0.000	1.000	0.062	0.241	0.000	1.000
4.5- 5.5 million yen	0.096	0.295	0.000	1.000	0.059	0.235	0.000	1.000
5.5- 7.5 million yen	0.136	0.343	0.000	1.000	0.074	0.261	0.000	1.000
7.5- 10 million yen	0.097	0.296	0.000	1.000	0.055	0.228	0.000	1.000
More than 10 million yen	0.054	0.226	0.000	1.000	0.055	0.228	0.000	1.000
Marital status (=1 if married)	0.814	0.389	0.000	1.000	0.698	0.459	0.000	1.000
Number of housemates (Over 20)	1.793	1.133	0.000	7.000	1.666	1.128	0.000	7.000
Number of housemates (Under 20)	0.628	0.978	0.000	6.000	0.672	0.983	0.000	5.000
Residence (in the 13 largest cities)	0.179	0.384	0.000	1.000	0.205	0.404	0.000	1.000
Residence (Other cities)	0.585	0.493	0.000	1.000	0.584	0.493	0.000	1.000
Real GDP (billion yen, in 2000 price) ^5)^	51.562	1.751	50.162	55.228	50.870	1.306	50.162	55.228
Unemployment rate (%) ^6)^	4.870	0.393	4.133	5.358	4.865	0.275	4.133	5.358
Number of Observations	4367				2970			

Note: (1) Japanese yen in 2005 price.

(2) Source from *The Retail Price Survey*, the Ministry of Internal Affairs and Communications.

(3) Source from *National Tax Agency Annual Statistical Report*, the National Tax Agency.

(4) Source from *Systems of the Local Taxation*, the Ministry of Internal Affairs and Communications.

(5) Source from *The National Accounts of Japan*, the Cabinet Office, the Government of Japan.

(6) Source from *the Labour Force Survey*, the Ministry of Internal Affairs and Communications.

### Empirical results and discussion

Table 3 presents the empirical results of equation (1) by the Probit and IV-Probit models^o^. The statistics of the Wald test for the exogeneity of the instrumented variables in the basic model are significant, which means that cigarette prices are statistically endogenous in the basic models. In addition, the first-stage F-statistics are sufficiently larger than 10, which means that the cigarette tax per pack has sufficient explanatory power for cigarette prices per pack. These results indicate that the IV-Probit model is appropriate to estimate the basic models, and the regular Probit model is appropriate to estimate the dynamic models.

**Table 3:** The effects on smoking participation

**Gender**	**Male**				**Female**			
**Model**	**Basic model**		**Dynamic model**		**Basic model**		**Dynamic model**	
Estimation method	Probit	IV-Probit	Probit	IV-Probit	Probit	IV-Probit	Probit	IV-Probit
Variables	Coef/SE	Coef/SE	Coef/SE	Coef/SE	Coef/SE	Coef/SE	Coef/SE	Coef/SE
Cigarette price per pack	-0.032**	-0.027**	-0.018	-0.026*	-0.090***	-0.086***	-0.059***	-0.071***
	(0.013)	(0.014)	(0.015)	(0.016)	(0.017)	(0.017)	(0.020)	(0.025)
Marginal effects	[-0.012]	[-0.010]	[-0.007]	[-0.009]	[-0.021]	[-0.020]	[-0.014]	[-0.016]
Health Promotion Law	-0.179	-0.186	-0.420***	-0.368**	-0.271	-0.263	-0.514**	-0.422
	(0.114)	(0.115)	(0.145)	(0.143)	(0.264)	(0.209)	(0.240)	(0.257)
Marginal effects	[-0.065]	[-0.067]	[-0.152]	[-0.133]	[-0.063]	[-0.055]	[-0.119]	[-0.080]
Age	-0.020***	-0.020***	-0.020***	-0.020***	-0.020***	-0.020***	-0.020***	-0.020***
	(0.002)	(0.002)	(0.002)	(0.002)	(0.003)	(0.003)	(0.003)	(0.003)
Years of education	-0.032***	-0.033***	-0.032***	-0.032***	-0.081***	-0.081***	-0.079***	-0.079***
	(0.007)	(0.007)	(0.007)	(0.007)	(0.018)	(0.016)	(0.017)	(0.016)
Office worker	0.006	0.006	0.007	0.008	0.185**	0.185**	0.176**	0.177**
	(0.089)	(0.088)	(0.089)	(0.089)	(0.078)	(0.072)	(0.079)	(0.072)
Manual worker	0.208**	0.209**	0.216**	0.216**	0.259**	0.261**	0.237**	0.241*
	(0.093)	(0.093)	(0.094)	(0.094)	(0.117)	(0.123)	(0.120)	(0.123)
Unemployment	0.238*	0.240*	0.240*	0.239*	0.461*	0.453*	0.437*	0.438*
	(0.133)	(0.132)	(0.134)	(0.133)	(0.260)	(0.253)	(0.262)	(0.253)
Income: 1–2.5 million yen	0.187*	0.187*	0.186*	0.187*	-0.079	-0.078	-0.077	-0.079
	(0.110)	(0.110)	(0.110)	(0.110)	(0.067)	(0.098)	(0.070)	(0.097)
Income: 2.5- 3.5 million yen	0.154	0.153	0.153	0.153	0.047	0.046	0.042	0.042
	(0.128)	(0.128)	(0.129)	(0.129)	(0.121)	(0.113)	(0.125)	(0.113)
Income: 3.5- 4.5 million yen	0.154	0.155	0.151	0.151	-0.111	-0.111	-0.109	-0.110
	(0.115)	(0.114)	(0.115)	(0.115)	(0.128)	(0.129)	(0.130)	(0.129)
Income: 4.5- 5.5 million yen	0.216*	0.216*	0.212*	0.213*	-0.040	-0.038	-0.055	-0.054
	(0.111)	(0.111)	(0.111)	(0.110)	(0.120)	(0.131)	(0.121)	(0.131)
Income: 5.5- 7.5 million yen	0.042	0.041	0.040	0.040	0.088	0.088	0.075	0.077
	(0.105)	(0.105)	(0.106)	(0.106)	(0.102)	(0.120)	(0.102)	(0.120)
Income: 7.5- 10 million yen	0.143	0.145	0.145	0.143	-0.032	-0.032	-0.049	-0.047
	(0.114)	(0.114)	(0.114)	(0.114)	(0.204)	(0.136)	(0.202)	(0.136)
Income: More than 10 million yen	-0.001	-0.001	0.002	0.001	-0.020	-0.018	-0.024	-0.027
	(0.104)	(0.103)	(0.103)	(0.103)	(0.144)	(0.141)	(0.145)	(0.141)
Marital status	-0.142***	-0.143***	-0.142***	-0.141***	-0.150**	-0.151**	-0.149**	-0.149**
	(0.053)	(0.053)	(0.053)	(0.053)	(0.061)	(0.068)	(0.062)	(0.068)
Number of housemates (Over 20)	0.002	0.002	0.002	0.002	-0.066**	-0.067**	-0.068**	-0.068**
	(0.020)	(0.020)	(0.020)	(0.020)	(0.029)	(0.028)	(0.029)	(0.028)
Number of housemates (Under 20)	0.007	0.007	0.006	0.006	0.010	0.010	0.010	0.010
	(0.024)	(0.023)	(0.024)	(0.024)	(0.041)	(0.033)	(0.040)	(0.033)
Residence (Other cities)	0.142***	0.143***	0.155***	0.153***	-0.470***	-0.479	-0.470***	-0.475
	(0.026)	(0.026)	(0.023)	(0.024)	(0.112)	(0.359)	(0.123)	(0.359)
Residence (in the 13 largest cities)	0.520***	0.514***	0.506***	0.515***	0.969***	0.954	0.911***	0.912
	(0.046)	(0.046)	(0.047)	(0.046)	(0.056)	(0.682)	(0.059)	(0.682)
Real GDP (billion yen)	0.378***	0.348***	0.567***	0.575***	1.221***	1.189***	1.361***	1.360***
	(0.088)	(0.090)	(0.109)	(0.110)	(0.129)	(0.130)	(0.157)	(0.155)
Unemployment rate	0.464***	0.471***	0.295***	0.301***	1.855***	1.807***	1.714***	1.723***
	(0.102)	(0.103)	(0.087)	(0.086)	(0.251)	(0.172)	(0.167)	(0.139)
Constant	-12.526***	-12.260***	-24.504***	-23.076***	-49.391***	-48.443***	-63.025***	-60.265***
	(2.671)	(2.663)	(5.574)	(5.510)	(5.909)	(4.603)	(8.660)	(9.459)
atanh *ρ*		-0.047***		0.029				
		(0.018)		(0.024)				
ln*σ*		-0.978***		-0.314***				
		(0.006)		(0.008)				
Health Promotion Law	Dummy	Dummy	Elapsed years	Elapsed years	Dummy	Dummy	Elapsed years	Elapsed years
Number of observations	4367	4367	4367	4367	2970	2970	2970	2970
Log pseudolikelihood	-2762.417	-4686.603	-2760.478	-7583.357	-1239.715	(TSE)	-1238.328	(TSE)
Wald test for H_0_: all coefficients = 0	χ^2^ (47) = 4.7e+10***	χ^2^ (41) = 8.4e+10***	χ^2^ (47) = 7.3e+10***	χ^2^ (40) = 3.1e+09***	χ^2^ (47) = 1.3e+10***	χ^2^ (124) = 599.16***	χ^2^ (47) = 1.1e+10***	χ^2^ (124) = 612.53***
Wald test for H_0_: local effects = 0	χ^2^ (43) = 1.1e+11***	χ^2^ (41) = 8.7e+10***	χ^2^ (44) = 2.9e+11***	χ^2^ (40) = 9.4e+10***	χ^2^ (44) = 2.3e+10***	χ^2^ (46) = 28.28	χ^2^ (45) = 2.6e+10***	χ^2^ (46) = 27.17
Wald test for H_0_: local effects * Other city = 0	χ^2^ (42) = 9.6e+10***	χ^2^ (41) = 8.1e+10***	χ^2^ (42) = 9.3e+10***	χ^2^ (40) = 2.9e+09***	χ^2^ (45) = 2.6e+10***	χ^2^ (46) = 28.23	χ^2^ (46) = 1.1e+11***	χ^2^ (46) = 27.47
Wald test for H_0_: local effects * the 13 largest cities = 0	χ^2^ (12) = 7199.49***	χ^2^ (12) = 7109.37***	χ^2^ (12) = 7294.49***	χ^2^ (12) = 7634.66***	χ^2^ (11) = 935.24***	χ^2^ (11) = 8.02	χ^2^ (11) = 864.79***	χ^2^ (11) = 7.66
Wald test for exogeneity of cigarette tax		χ^2^ (1) = 7.21***		χ^2^ (1) = 1.47		χ^2^ (1) = 4.00**		χ^2^ (1) = 1.23
First stage F-statistics		χ^2^ (1) = 137789.44***		χ^2^ (1) = 11787.44***		χ^2^ (1) = 45654.87***		χ^2^ (1) = 13169.86***

Note: (1) Robust standard errors allowing for correlated residuals within prefectures are shown in parentheses.

(2) ***, **, and * represent statistical significance at the 1, 5, and 10 percent levels, respectively.

(3) All equations include the prefectural dummy variables and the interactions between prefectural dummies and residence dummy variables.

(4) Marginal effects in squared parentheses are evaluated at the sample mean.

(5) TSE indicates that Newey’s [31] two-step estimation is used.

An increase in cigarette prices has a statistically significant negative effect on smoking probability for both genders. Specifically, an increase in cigarette prices per pack of 1 yen reduces the smoking participation of males by 1.0 percent and that of females by 1.4 to 2.0 percent^f^. These values are higher than the values of -0.61 (males) and -0.46 (females) found by Kadoda et al. [5] and the values of -0.5 percent (males) and -0.1 percent (females) found by Kamimura and Noda [11]. The reason for these differences in marginal effects is that Kadoda et al. [5] and Kamimura and Noda [11] do not include the enforcement of the HPL and trend variables in their empirical equations. I also find that the diffusion effect of the HPL has a statistically significant negative effect on the smoking probability of both genders. These findings are unlike the results of Ishii and Kawai [9] using single-year cross-sectional data. The HPL has a statistically significant effect on reducing the smoking participation of males by 15.2 percent and of females by 11.9 percent. The result that these smoking control policies have a statistically significant negative effect on smoking behavior among females seems to contradict the findings of the macro statistics in Figure 1, which remain almost constant. This contradiction may be caused by the higher smoking rates of the sample, as mentioned above. For the other explanatory variables, older, more highly educated, and married individuals have a statistically significant lower smoking probability for both genders. Manual workers, the unemployed, and female office workers tend to smoke more cigarettes than non-workers. Females with housemates aged 20 years and older have a statistically significant lower probability of smoking. People who live in the 13 largest cities have a statistically significant higher probability of smoking. Males living in the other cities also have a statistically significant higher probability of smoking, but females have a statistically significant lower probability of smoking.

Table 4 shows the effects of smoking control policies on each employment format. The statistics of the Wald test for the exogeneity of the instrumented variables in the basic and dynamic model for males are significant, which means that cigarette prices are statistically endogenous in these models. In addition, all of the first-stage F-statistics substantially exceed 10, which means that cigarette taxes per pack have sufficient explanatory power for cigarette prices per pack. These results indicate that the IV-Probit model is appropriate to estimate the models for males, and the regular Probit model is appropriate for females.

**Table 4:** The effects on smoking participation by employment status

**Gender**	**Male**				**Female**			
**Model**	**Basic model**		**Dynamic model**		**Basic model**		**Dymanic model**	
Estimation method	Probit	IV-Probit	Probit	IV-Probit	Probit	IV-Probit	Probit	IV-Probit
Variables	ME/SE	ME/SE	ME/SE	ME/SE	ME/SE	ME/SE	ME/SE	ME/SE
Cigarette tax per pack	-0.036	-0.029***	-0.008	0.007	-0.079***	-0.073***	-0.008	-0.044
* Office worker	(0.014)	(0.011)	(0.021)	(0.023)	(0.020)	(0.020)	(0.037)	(0.044)
Marginal effects	[-0.013]	[-0.012]	[-0.003]	[0.003]	[-0.018]	[-0.017]	[-0.002]	[-0.010]
Cigarette tax per pack	-0.032**	-0.024*	-0.032*	-0.034	-0.034	-0.004	-0.217	-0.243*
* Manual worker	(0.014)	(0.013)	(0.020)	(0.033)	(0.029)	(0.050)	(0.144)	(0.138)
Marginal effects	[-0.011]	[-0.010]	[-0.012]	[-0.014]	[-0.008]	[-0.001]	[-0.050]	[-0.050]
Cigarette tax per pack	-0.002	0.037	0.140*	0.086	-0.151**	-0.161	-0.434*	-0.527
* Unemployment	(0.034)	(0.049)	(0.081)	(0.111)	(0.062)	(0.099)	(0.254)	(1.490)
Marginal effects	[-0.001]	[0.015]	[0.050]	[0.034]	[-0.035]	[-0.035]	[-0.100]	[-0.092]
Cigarette tax per pack	-0.030**	-0.028**	-0.034	-0.079***	-0.099***	-0.097***	-0.086***	-0.073*
* Non-worker	(0.013)	(0.012)	(0.024)	(0.028)	(0.016)	(0.019)	(0.028)	(0.044)
Marginal effects	[-0.011]	[-0.011]	-[0.012]	[-0.031]	[-0.023]	[-0.022]	[-0.020]	[-0.017]
the Health Promotion Law	-0.024	-0.053	-0.483***	-0.608***	-0.362	-0.375	-0.896**	-0.611
* Office worker	(0.150)	(0.136)	(0.162)	(0.218)	(0.320)	(0.294)	(0.350)	(0.381)
Marginal effects	[-0.009]	[-0.021]	[-0.174]	[-0.233]	[-0.084]	[-0.072]	[-0.206]	[-0.101]
the Health Promotion Law	-0.289	-0.338	-0.331*	-0.324	-1.178**	-1.576**	0.792	0.988
* Manual worker	(0.181)	(0.218)	(0.197)	(0.283)	(0.467)	(0.783)	(1.136)	(1.076)
Marginal effects	[-0.104]	[-0.134]	[-0.119]	[-0.128]	[-0.272]	[-0.149]	[0.183]	[0.332]
the Health Promotion Law	-0.336	-0.832	-1.417**	-1.000	-0.103	0.160	2.029	2.795
* Unemployment	(0.553)	(0.733)	(0.669)	(0.817)	(1.294)	(1.664)	(1.946)	(13.929)
Marginal effects	[-0.121]	[-0.306]	[-0.511]	[-0.354]	[-0.024]	[0.041]	[0.468]	[0.809]
the Health Promotion Law	-0.387***	-0.301*	-0.327	0.006	-0.045	0.024	-0.345	-0.439
* Non-worker	(0.141)	(0.175)	(0.210)	(0.249)	(0.315)	(0.298)	(0.302)	(0.380)
Marginal effects	[-0.140]	[-0.119]	[-0.118]	[0.002]	[-0.010]	[0.006]	[-0.080]	[-0.081]
Age	-0.020***	-0.020***	-0.020***	-0.020***	-0.021***	-0.021***	-0.020***	-0.020***
	(0.002)	(0.002)	(0.002)	(0.002)	(0.003)	(0.003)	(0.003)	(0.003)
Years of education	-0.032***	-0.032***	-0.032***	-0.032***	-0.082***	-0.083***	-0.080***	-0.080***
	(0.007)	(0.008)	(0.007)	(0.008)	(0.018)	(0.016)	(0.017)	(0.016)
Office worker	1.438	0.126	-6.243	-20.460**	-4.575	-5.484	-18.674	-6.883
	(1.733)	(2.150)	(5.961)	(9.133)	(2.993)	(4.014)	(12.057)	(16.847)
Manual worker	0.678	-0.884	-0.209	-10.363	-15.282**	-21.916*	31.434	40.728
	(2.021)	(2.779)	(6.713)	(10.694)	(7.366)	(11.323)	(35.158)	(35.187)
Unemployment	-6.472	-15.516	-41.446*	-39.324	13.068	15.864	83.559	108.994
	(6.984)	(11.305)	(21.691)	(27.512)	(13.809)	(23.441)	(60.593)	(355.841)
Income: 1–2.5 million yen	0.188*	0.190	0.183*	0.182	-0.083	-0.084	-0.070	-0.073
	(0.110)	(0.116)	(0.110)	(0.116)	(0.068)	(0.098)	(0.073)	(0.098)
Income: 2.5- 3.5 million yen	0.153	0.154	0.148	0.149	0.045	0.042	0.044	0.043
	(0.128)	(0.114)	(0.129)	(0.114)	(0.125)	(0.114)	(0.126)	(0.114)
Income: 3.5- 4.5 million yen	0.154	0.156	0.145	0.144	-0.110	-0.109	-0.108	-0.108
	(0.115)	(0.113)	(0.116)	(0.113)	(0.128)	(0.129)	(0.131)	(0.129)
Income: 4.5- 5.5 million yen	0.214*	0.215*	0.207*	0.208*	-0.053	-0.052	-0.082	-0.079
	(0.111)	(0.116)	(0.110)	(0.116)	(0.120)	(0.132)	(0.123)	(0.133)
Income: 5.5- 7.5 million yen	0.039	0.039	0.033	0.033	0.075	0.073	0.058	0.065
	(0.107)	(0.110)	(0.106)	(0.110)	(0.103)	(0.122)	(0.101)	(0.122)
Income: 7.5- 10 million yen	0.140	0.143	0.140	0.143	-0.045	-0.046	-0.060	-0.054
	(0.114)	(0.114)	(0.115)	(0.114)	(0.204)	(0.138)	(0.197)	(0.138)
Income: More than 10 million yen	0.001	0.002	-0.001	-0.001	-0.034	-0.031	-0.044	-0.043
	(0.105)	(0.126)	(0.103)	(0.126)	(0.150)	(0.143)	(0.150)	(0.142)
Marital status	-0.141**	-0.141**	-0.140**	-0.139**	-0.149**	-0.150**	-0.148**	-0.147**
	(0.053)	(0.061)	(0.054)	(0.061)	(0.062)	(0.068)	(0.062)	(0.068)
Number of housemates (Over 20)	0.002	0.003	0.002	0.002	-0.067**	-0.068**	-0.069**	-0.069**
	(0.020)	(0.019)	(0.020)	(0.019)	(0.030)	(0.028)	(0.030)	(0.028)
Number of housemates (Under 20)	0.007	0.007	0.006	0.005	0.011	0.011	0.009	0.010
	(0.024)	(0.023)	(0.024)	(0.023)	(0.040)	(0.033)	(0.039)	(0.033)
Residence (Other cities)	0.148***	0.154	0.155***	0.157	-0.475***	-0.491	-0.492***	-0.497
	(0.025)	(0.252)	(0.023)	(0.252)	(0.111)	(0.361)	(0.135)	(0.360)
Residence (in the 13 largest cities)	0.542***	0.579	0.591***	0.539	0.965***	0.936	0.898***	0.914
	(0.055)	(0.460)	(0.069)	(0.468)	(0.068)	(0.687)	(0.065)	(0.677)
Real GDP (billion yen)	0.384***	0.351***	0.566***	0.575***	1.204***	1.165***	1.375***	1.373***
	(0.089)	(0.073)	(0.109)	(0.110)	(0.131)	(0.132)	(0.161)	(0.156)
Unemployment rate	0.462***	0.475***	0.288***	0.294***	1.861***	1.810***	1.730***	1.747***
	(0.101)	(0.094)	(0.087)	(0.085)	(0.253)	(0.173)	(0.168)	(0.141)
Constant	-13.374***	-12.069***	-20.725***	-10.466	-46.296***	-44.541***	-57.252***	-60.340***
	(2.969)	(2.749)	(7.210)	(8.369)	(6.332)	(5.130)	(10.610)	(12.844)
Health Promotion Law	Dummy	Dummy	Elapsed years	Elapsed years	Dummy	Dummy	Elapsed years	Elapsed years
Number of observations	4367	4367	4367	4367	2970	2970	2970	2970
Log pseudolikelihood	-2757.817	(TSE)	-2756.472	(TSE)	-1235.905	(TSE)	-1232.920	(TSE)
Wald test for H_0_: all coefficients = 0	χ^2^ (47) = 4.2e+10***	χ^2^ (131) = 469.41***	χ^2^ (47) = 5.3e+10***	χ^2^ (131) = 475.15***	χ^2^ (47) = 9.6e+09***	χ^2^ (130) = 601.47***	χ^2^ (47) = 7.8e+09***	χ^2^ (130) = 613.52***
Wald test for H_0_: local effects = 0	χ^2^ (44) = 3.0e+11***	χ^2^ (46) = 33.11	χ^2^ (41) = 6.7e+10***	χ^2^ (46) = 33.06	χ^2^ (46) = 2.7e+10***	χ^2^ (46) = 28.48	χ^2^ (45) = 5.3e+10***	χ^2^ (46) = 26.87
Wald test for H_0_: local effects * Other city = 0	χ^2^ (42) = 9.1e+10***	χ^2^ (46) = 29.32	χ^2^ (43) = 7.3e+10***	χ^2^ (46) = 30.05	χ^2^ (45) = 2.1e+10***	χ^2^ (46) = 27.72	χ^2^ (45) = 3.9e+10***	χ^2^ (46) = 26.88
Wald test for H_0_: local effects * the 13 largest cities = 0	χ^2^ (12) = 4645.32***	χ^2^ (12) = 6.19	χ^2^ (12) = 4362.10***	χ^2^ (12) = 6.63	χ^2^ (11) = 456.15***	χ^2^ (11) = 8.15	χ^2^ (11) = 858.46***	χ^2^ (11) = 8.24
Wald test for exogeneity of cigarette taxes: χ^2^ (1)		χ^2^ (4) = 9.21*		χ^2^ (4) = 7.95*		χ^2^ (4) = 5.73		χ^2^ (4) = 2.33
First stage F-stat								
for CPI * Office worker		F(4,4281) = 5017.10***		F(4,4281) = 1907.96***		F(4,2885) = 2445.51***		F(4,2885) = 1219.26***
for CPI * Manual worker		F(4,4281) = 4337.32***		F(4,4281) = 1601.43***		F(4,2885) = 1436.00***		F(4,2885) = 786.06***
for CPI * Unemployment		F(4,4281) = 1544.91***		F(4,4281) = 1812.30***		F(4,2885) = 1890.17***		F(4,2885) = 952.44***
for CPI * Nonworker		F(4,4281) = 4543.63***		F(4,4281) = 1684.49***		F(4,2885) = 2718.65***		F(4,2885) = 1194.51***

Note: (1) Robust standard errors allowing for correlated residuals within prefectures are shown in parentheses.

(2) ***, **, and * represent statistical significance at the 1, 5, and 10 percent levels, respectively.

(3) All equations include the prefectural dummy variables and the interactions between prefectural dummies and residence dummy variables.

(4) Marginal effects in squared parentheses are evaluated at the sample mean.

(5) TSE indicates that Newey’s [31] two-step estimation is used.

An increase in cigarette prices has a particularly significant and negative effect on smoking probability for non-workers. Specifically, an increase in the cigarette price per pack of 1 yen has a statistically significant effect on reducing the smoking probability of male non-workers by 1.1 to 3.1 percent and that of female non-workers by 2.0 to 2.3 percent.

An increase in cigarette prices also statistically significantly reduces the smoking probabilities of office workers by 1.2 percent for males and 1.8 percent for females, male manual workers by 1.0 percent, and female unemployed people by 2.0 to 2.3 percent. I also find that the diffusion effect of the HPL has a statistically significantly large negative effect on the smoking probability of office workers; it reduces the smoking probability of males by 21.8 percent and that of females by 20.6 percent. The introduction of the HPL also has a negative impact on the smoking probabilities of female manual workers by 27.2 percent and of male non-workers by 11.9 percent. The estimation results of the coefficients of the other explanatory variables are similar to those in Table 3.

## Conclusions

This article comprehensively examines the impact of an increase in cigarette taxes and the enforcement of the HPL on individuals’ smoking choices. The empirical results show that an increase in cigarette prices has a statistically significant effect on reducing the smoking probability of males by 1.1 percent and that of females by 1.3 to 1.9 percent. The enforcement of the HPL has a statistically significant effect on reducing the smoking probability of males by 13.2 percent and that of females by 13.1 percent. Furthermore, an increase in cigarette prices has a statistically significant negative effect on the smoking probability of office workers, non-workers, male manual workers, and female unemployed people, and the enforcement of the HPL has a statistically significant effect on decreasing the smoking probabilities of office workers and female manual workers.

Nevertheless, the percentage of Japanese male smokers is much higher than that of the other developed countries. Therefore, the Japanese government will continue to establish and evaluate smoking control policies, as stated in *Health Japan 21*. Although the empirical results of this study indicate that recent smoking control policies in Japan have contributed to decreasing smoking rates, there is room for improvement in the HPL. For example, penalties enforceable by managers of public spaces would prevent Japanese smokers from smoking.

Finally, this study has three important limitations. First, it is difficult to completely distinguish between the effects of smoking control policies and time trends because the Japanese smoking control policies examined in this study were uniformly enforced nationwide at a certain time. The convenient approach in this study can partially capture those effects, and there is a possibility that the estimators may be biased because of the omitted variable bias. Using a natural experiment, such as the introduction of *Taspo* cards (where the timing of the introduction varied by region; Kanda et al. [23]) could overcome this problem. Second, there is a possibility that smoking control policies may affect the volume of smokers’ cigarette consumption as well as smoking intensity, but these data are not available from the JGSS. Third, the following factors that affect smoking choices were not taken into consideration: an individual’s smoking history or extent of nicotine addiction and preferences for risk and time. The parameters in this study are biased if the above factors and any of the explanatory variables are correlated. The solutions to these limitations represent important research challenges for future studies.

## Endnotes

^a^See the WHO Regional Office for Europe [24].

^b^In addition, Kamimura and Noda [11], Ii and Ohkusa [25], and Ida and Goto [26,27] find that individuals with higher relative risk-aversion coefficients significantly reduce the demand for cigarettes and the probability of smoking. Kanda et al. [23] examines the effect of the introduction of an age-verification system for tobacco purchase through *Taspo* on minors’ demand for cigarettes. Yuda [28] examines the impact of recent smoking control policies on individuals’ level of subjective happiness.

^c^The other smoking control policies implemented in 2000 to 2006 are smoking bans in the street enforced in some municipalities and the enlargement of health warnings on cigarette packages. With regard to the former policies, several local governments and railroad companies have voluntarily taken measures to prevent second-hand smoke inhalation, including levying fines for smoking in public spaces (for example, see Ueda et al. [12]). The latter policy is that the Japanese government required cigarette companies to enlarge the warning labels printed on both sides of the package to comply with the WHO’s convention in July 2005. Specifically, cigarette companies are obliged to print warnings, such as health risks due to smoking, the risk of nicotine addiction, and the risk of premature birth, on more than 30 percent of the front and back of the packages.

^d^See the JGSS website: http://jgss.daishodai.ac.jp/english/index.html

^e^As introduced in Table 1 and the website of the JGSS Research Center (http://jgss.daishodai.ac.jp/english/index.html), the JGSS datasets for 2000, 2001, 2002, 2003, 2005, 2006, and 2008 are available. This study does not use the newest JGSS data for 2008 because I cannot take into consideration the effect of the introduction of new cigarette vending machines with built-in age verifiers using IC cards, known as “*Taspo*”, in 2008, which affect smoking behavior. Because the information on whether a respondent has a *Taspo* is not available from the JGSS, I cannot appropriately treat the omitted variable bias caused by not being able to add a dummy variable of having a *Taspo* on the empirical equations. Moreover, I cannot appropriately treat the selection biases caused by adding some regressors that partially capture the effect of having a *Taspo* (for example, the *Taspo* holding ratios at the prefectural level, if they are available) because having a *Taspo* or not is not randomly assigned.

^f^The respondents are also asked, “Have you ever tried to give up smoking?” with a choice of either “Yes” or “No.”

^g^Akiyama et al. [14] note that smoking rates from the National Nutrition and Health Survey are under-estimated because of these differences.

^h^As mentioned in footnote C, the Japanese government implemented the enlargement of health warnings on cigarette packages. However, this paper does not examine this policy effect because the Subcommittee on the Tobacco Industries of the Fiscal System Council [29] shows that enlargement of the health warnings on cigarette packages has little effect on quitting smoking.

^i^The four employment formats are office workers, manual workers, the unemployed, and non-workers. A more detailed explanation is provided in footnote M.

^j^Income is defined as the respondent’s pretax family income in the previous year if the main income source of the respondent is the spouse, parents, or other family members. Otherwise, income is defined as the respondent’s pretax income in the previous year.

^k^It is difficult to completely distinguish between the effects of smoking control policies and time trends (yearly effects) in this empirical equation (1) because the Japanese smoking control policies examined in this study were uniformly enforced nationwide at a certain time. However, if I do not add the trend variables as independent variables in the empirical equations, these effects may be absorbed into the effects of cigarette prices and the enforcement of the HPL, which biases the estimators of the policies.

^l^This convenient approach can be considered to partially estimate those effects. If this strategy is not employed, the estimators may be biased because of the omitted variable bias.

^m^According to The Japan Institute for Labor Policy and Training [30], *manual workers* are defined as employees whose main workplace is neither a private office nor a public place, such as collectors, street vendors, peddlers, delivery people, routemen, street and door-to-door salespeople, news vendors, garbage collectors, insurance agents, insurance brokers, insurance underwriters, childcare workers (private household), cooks (private household), housekeepers (private household), laundresses (private household), maids, servants (private household), farm foremen, farm laborers, gardeners, groundskeepers, stock farmers, foresters, fishermen, oyster farmers, taxi drivers, chauffeurs, truck drivers, teamsters, mail carriers, mail handlers, messengers, mining engineers, mine workers, coal miners, rock carvers, electric power line workers, cable workers, plasterers, plumbers, pipe fitters, bricklayers, stonemasons, civil engineers, road engineers, railroad engineers, foremen, crane operators, derrick operators, hoist operators, chainmen, road workers, construction laborers, millwrights, and carpenters. *Office workers* are workers other than *manual workers*. *The unemployed* consists of unemployed persons, and *Non-employed people* consists of individuals who are both unemployed and not in the labor force.

^n^Because the maximum likelihood estimator (MLE) may have difficulty converging, especially with multiple endogenous variables, I estimate Newey’s [31] minimum chi-squared estimator (two-step estimator), which is less efficient than the MLE. Although the estimated coefficients from the two models are not directly comparable, the two-step estimates can be used to test for statistically significant relationships (Stata Corporation [32], Wooldridge [33]).

^o^The empirical equation (1) for the female sample is estimated by the two-step estimation because the likelihood functions are not converged in the case of the maximum likelihood estimation.

^p^The marginal effect of an endogenous variable *y* in the two-step IV-Probit model is obtained as follows:

(none2) $$\Phi \left[x^{o}\beta +\alpha \left(y^{o}+1\right)\right]-\Phi \left[x^{o}\beta +\alpha y^{o}\right]$$

where **x**^**o**^ includes the mean values of all independent variables except for *y* in equation (3), and *y*^*o*^ is the mean value of *y*. *α* and **β** are the estimated parameters of *y* and **x**, respectively (Wooldridge [33]).

## Abbreviations

GDP: Gross Domestic Product; HPL: Health Promotion Law; IV: Instrumental variable; JGSS: Japanese General Social Survey; JT: Japan Tobacco, Inc.; WHO: World Health Organization.

## Competing interests

The author declares that they have no competing interest.

## Authors’ contribution

The author conceived the study, undertook the analysis and did the write up of the manuscript. The author read and approved the final manuscript.
